# Correction to “Phenotypic and Genetic Characteristics of 24 Cases of Early Infantile Epileptic Encephalopathy in East China, Including a Rare Case of Biallelic UGDH Mutations”

**DOI:** 10.1002/mgg3.70145

**Published:** 2025-09-24

Jiang, L., S. Bi, L. Lin, F. He, and F. Deng. 2023. “Phenotypic and Genetic Characteristics of 24 Cases of Early Infantile Epileptic Encephalopathy in East China, Including a Rare Case of Biallelic UGDH Mutations.” *Molecular Genetics & Genomic Medicine* 11, no. 12: e2269. 10.1002/mgg3.2269.

Correction to the Abstract section

In the Abstract section, under “Methods,” the text “This study retrospectively included the gene variants from 24 EIEE‐positive patients admitted between January 2021 and January 2022 to a hospital in Anhui Province, China.” was incorrect. This should have read as “This study retrospectively included the gene variants from 24 EIEE‐positive patients admitted between January 2018 and December 2022 to a hospital in Anhui Province, China.”

Correction to Section 2.1 Study group

In Section 2 DATA AND METHODS section, under “2.1 Study group,” the text “Between January 2017 and August 2020, we reviewed 24 EIEE cases without known causes in the Department of Neurology of Anhui Children's Hospital.” was incorrect. This should have read as “Between January 2018 and December 2022, we reviewed 24 EIEE cases without known causes in the Department of Neurology of Anhui Children's Hospital.”

We apologize for this error.

